# Capsule Design for Blue Light Therapy against *Helicobacter pylori*

**DOI:** 10.1371/journal.pone.0147531

**Published:** 2016-01-27

**Authors:** Zhangyong Li, Binbin Ren, Haiyan Tan, Shengrong Liu, Wei Wang, Yu Pang, Jinzhao Lin, Chen Zeng

**Affiliations:** 1 Research Center of Biomedical Engineering, Chongqing University of Posts and Telecommunications, Chongqing, China; 2 Department of Physics, the George Washington University, Washington, D. C., United States of America; Indian Institute of Science, INDIA

## Abstract

A photo-medical capsule that emits blue light for *Helicobacter pylori* treatment was described in this paper. The system consists of modules for pH sensing and measuring, light-emitting diode driver circuit, radio communication and microcontroller, and power management. The system can differentiate locations by monitoring the pH values of the gastrointestinal tract, and turn on and off the blue light according to the preset range of pH values. Our experimental tests show that the capsule can operate in the effective light therapy mode for more than 32 minutes and the wireless communication module can reliably transmit the measured pH value to a receiver located outside the body.

## Introduction

Recently, blue light is widely used in clinic to kill bacteria. It is reported that blue light exhibits bactericidal effects against cultured *Helicobacter pylori (H*. *pylori)* and many other microbial bacteria around the wavelength of 405nm [1]. One hypothesis on the killing mechanism suggests that the bacteria probably accumulate photoactive porphyrins [2]. *H*. *pylori* colonizes the mucus layer of the human stomach and causes peptic ulcers and adenocarcinoma [3]. The traditional treatments use drugs to kill *H*. *pylori*, but inappropriate drug usage could pose a threat to public health because the bacteria could become drug-resistant [4]. It is thus beneficial to supplement the traditional method with blue-light therapy. Early experiments on the effect of blue light on the cultured *H*. *pylori* bacteria *in vitro* were performed in Ref. [1]. Additional tests indicated that the largest reduction in bacterial load was in the antrum (>97%), followed by body (>95%) and fundus (>86%), with a cycle treatment by blue light at the wavelength of 408±2nm [5]. These results proved that blue light could be used to kill *H*. *pylori* in clinic. To carry out this treatment in a noninvasive and economical manner, a micro device termed blue light therapy capsule is proposed in this paper.

Advancement in light-emitting diode (LED) and semiconductor technology has prompted highly integrated applications. Endoscopy capsule and physiological parameters detection capsule are just some representatives. As a typical micro medical device, the first endoscopy capsule named M2A by Given Imaging Corporation in 2001 provided a noninvasive detection method for digestive tract. Since then, there have been many different kinds of capsule devices developed by various institutes and companies, such as PillCam SB approved by the Food and Drug Administration [6]. Much of the current development on endoscopy capsule concentrates on intelligent control, wireless charging, and high-resolution video acquisition [7–9]. Different capsule devices have been designed for different purposes in clinical treatments such as non-invasive capsule for targeted drug delivery, capsule robot for local surgery, or monitoring capsule for physiological information collection of the digestive tract [10–14]. Overall, the capsule devices are shrinking in size, growing in functionalities, and becoming easier to use.

The objective of this paper is to propose a new therapeutic capsule for blue-light treatment against *H*. *pylori* regulated by pH monitoring. Eight blue LEDs at the wavelength of 408±2nm are the optical source with the radiation energy exceeding 140mW/cm^2^ [15]. The blue LEDs are driven by a constant current, and their power is controlled by the micro control unit (MCU) with pulse width modulated (PWM) signal. A pH sensor is used to measure pH values ranging from 1 to 9 for the whole digestive tract. More significantly, the pH value indicates the approximate location of the capsule when it moves along in the digestive tract. A measuring circuit unit is applied to amplify the pH voltage signal, suppress the noise, and improve the accuracy of the location estimation. A low power-consumption microcontroller is used to process the pH signal, control the system status, and generate PWM signal for LED driver. A wireless communication module is employed to send the measured pH values to the receiver.

## Materials and Methods

### System Architecture

**Fig 1:**
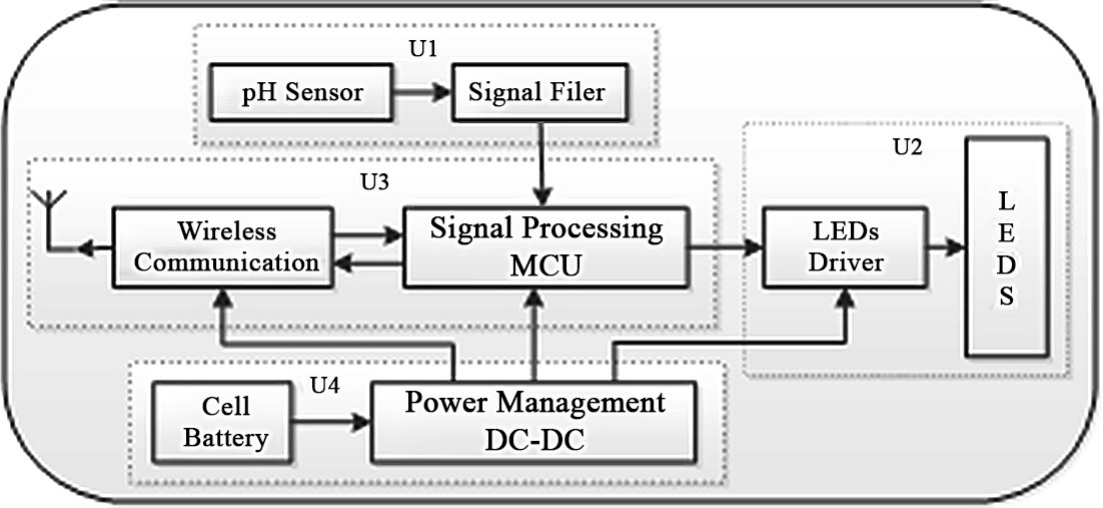
Architecture inside the capsule.

The inner architecture of the proposed capsule, shown in Fig 1, can be divided into four functional units. The first functional unit consists of a pH sensor and a measuring circuit to detect the pH value. The pH value is one of the most important physiological parameters of the digestive tract, and it can be used to trace the location and evaluate the digestive function. The pH sensor has two electrodes: the positive electrode measures the changes of voltage that reflects the changes of pH values, and the negative electrode serves as a reference input. The measuring circuit amplifies the voltage signal from the pH sensor so that the MCU can acquire the analog voltage signal accurately.

The second functional unit is the optical source composed of blue LEDs and LED driver circuit. Multiple LEDs instead of single-chip are designed with serial connection. The driving integrated circuit outputs stable current for the LEDs. Furthermore the output current can be regulated by PWM signal, which is generated by the MCU.

The wireless communication and microcontroller module form the third functional unit that facilitates information exchange among the receiver, MCU, LEDs driver, and pH sensor. The wireless communication module transmits pH value outside the body. The MCU can accordingly modulate the PWM of LEDs driver to change the current of LEDs or other system parameters. The MCU consists of the microcontroller and its peripheral components. The MCU processes the pH data to pinpoint the capsule’s location and turn on LEDs when the capsule moves into the stomach. The MCU also sends the control signal to the pH testing unit as well as the PWM signal to the optical source to conserve the power supply. The last significant unit of the system is for power supply. This unit contains a cell battery and a power management circuit. The circuit includes one DC-DC high efficient buck-boost convertor to supply the power for the system and one low-dropout regulator, which is controlled by the MCU, to supply the power for the pH detection unit.

### Design of System Units

#### The pH sensor and measuring circuit unit

The measuring range and resolution of pH sensor are the most significant parameters of the design. The pH values range from 1 to 7.5 for the whole digestive tract with a pH value about 7 in the esophagus, 1~4 for the gastric juice, and 6.6~7.5 for the intestinal juice [16–18]. Clearly, the sensor needs to cover the entire pH range with a resolution less than 2.6 (2.6 is the smallest difference in pH value between different digestive organ). Small sensor size is also required for the capsule integration. Because of the above considerations, antimony electrode is selected as the pH sensor whose detection range is from 1 to 9, sensitivity about 42±2mV/pH, and resolution better than 0.2 in pH value. The antimony electrode has a cylinder shape with 5.5mm in diameter and 7.0mm in length. The antimony electrode belongs to the class of oxidation-reduction solid electrode. The pH sensor has two electrodes, one for pH measurement and the other for reference. The voltage between the two electrodes is measured, and the change in voltage scales linearly with the change in pH value at a fixed ambient temperature.

#### LEDs and LED driver unit

**Fig 2:**
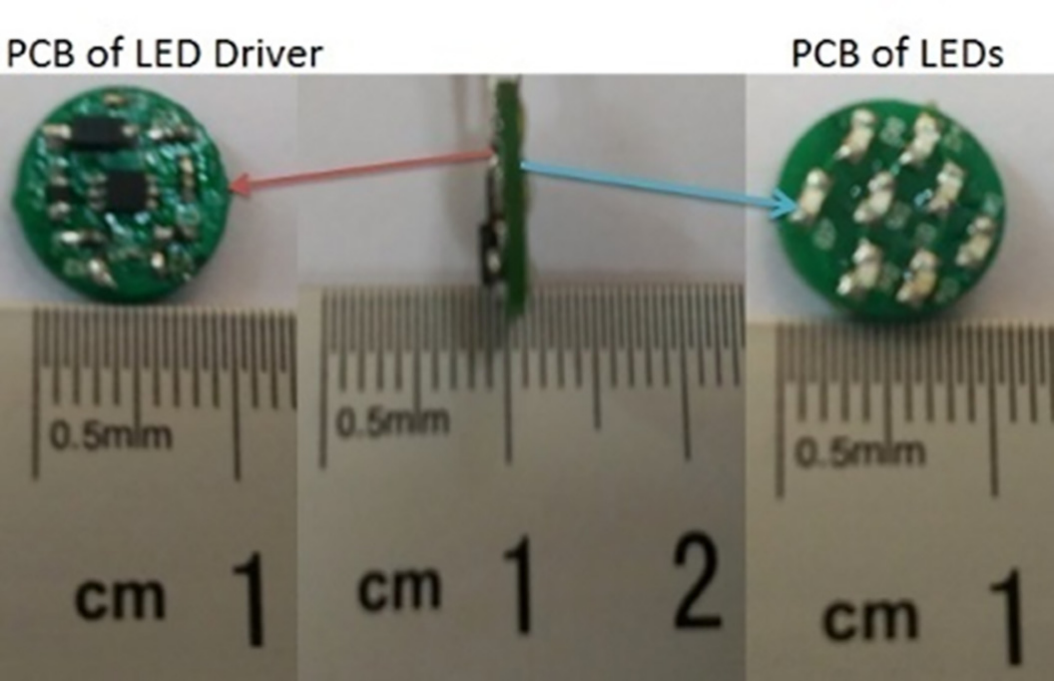
PCB LEDs and LED driver.

The InGaN LED with a wavelength of 408±2nm (Nichia Corp, Torrance, CA) was employed in this system. Given the same power cost, the multichip LED structure tends to produce more light and generate less thermal resistance than a single-chip LED in a limited space with a current drive employed [19–22]. There are eight LEDs in series, driven by constant current. As shown in Fig 2, the LEDs were distributed on a circular PCB board of only 11mm in diameter. The break-over voltage of the LED is 3.0V and the max drive current is 20mA. The current from LEDs’ driver controls the power of LEDs. Eq (1) reflects the relationship among the drive current *I*_*F*_, the external sensor resistor *R*_*set*_, and the feedback voltage *V*_*FB*_.

$$I_{F}=\frac{V_{FB}}{R_{set}}$$ (1)

The voltage *V*_*FB*_ varies linearly with the PWM duty cycle with a typical frequency. The control signal port of the driver chip is connected to the MCU so that the PWM signal can be applied to adjust the power of light for therapy. Fig 2 shows the PCB for LED drive circuit and LED light source.

#### Design of wireless communication and MCU unit

**Fig 3:**
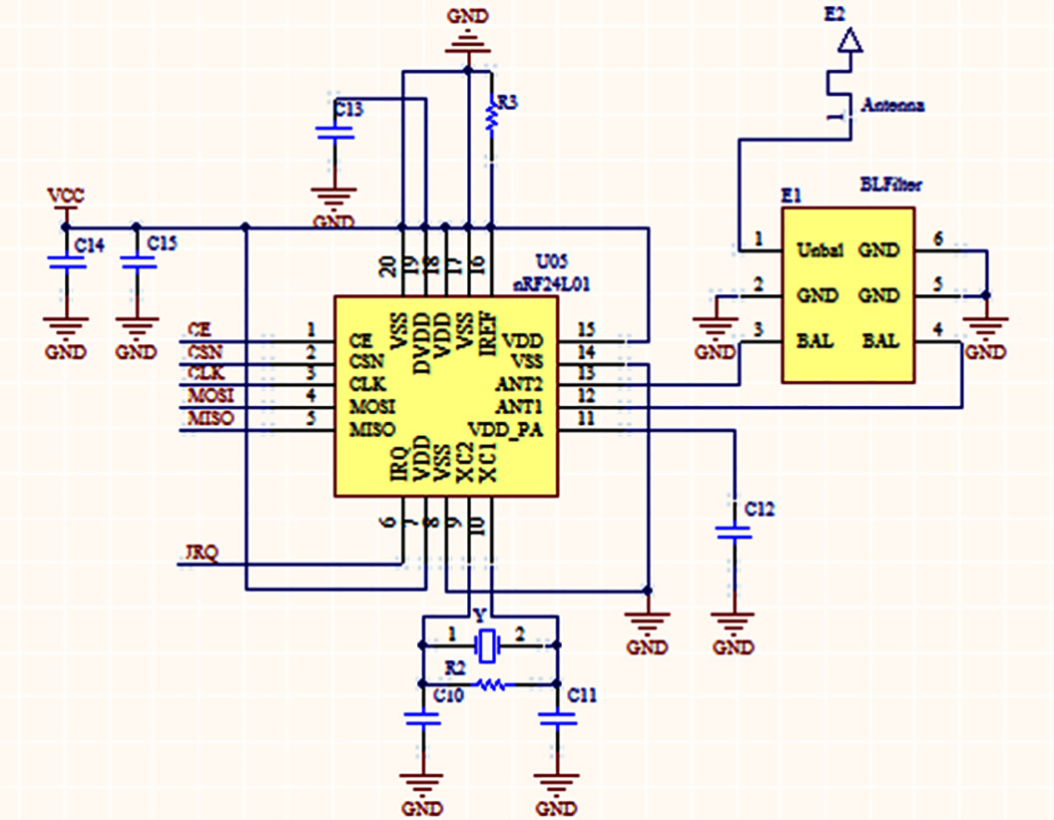
Circuit configuration of nRF24L01 at 2.4GHz.

The wireless communication handles information exchange. The wireless chip nRF24L01 (Nordic Semiconductor) is a single chip radio transceiver for the worldwide 2.4–2.5 GHz band. Current consumption is very low, only 9.0 mA in normal mode, 12.3 mA in receive mode, and 900nA in power down mode. With a few peripheral components within a 4*4 mm package, the RF chip is suitable for capsule design. The configuration of RF communication unit is shown in Fig 3.

MSP430G2203, produced by TI Corporation, is selected as the core component of the MCU module. It is a low power cost and mixed-signal controller with analog-to-digital converter, timer, and direct memory access all embedded on a single chip. The analog-to-digital converter registers are used to obtain the pH signal every ten seconds. And the Timer-A register generates the control signal. The data will be transmitted to the wireless communication module through serial peripheral interface, and finally sent to the receiver. The PWM signal is generated by the Timer-A too, and the PWM duty cycle can be adjusted to regulate the light based on the feedback information. The top priority of the MCU is to identify the capsule’s location according to the pH value measured and change the status of the LEDs respectively. Overall, the MCU swaps between wake-up mode and sleep to turn on and off the wireless communication module and pH testing units to save power.

#### Design of power unit

The power unit includes battery, magnetically controlled switch, and power management module. In this system, battery is the power source with limited energy. So an efficient power management is very important to prolong the system’s effective working time. Since the whole unit is contained within an anticorrosion shell with its electronic switches becoming inaccessible, magnetically controlled switch is employed as a master switch to save power before the capsule is ingested. When the capsule is in the magnetic cover, the switch is off and the capsule is inactive. Once the capsule is taken out of the magnetic cover and ingested, the switch is on and the capsule is activated. The power management module supplies stable and low noise voltage for the system. The high efficient buck-boost convertor TPS63001 from TI Corporation is selected as the power management chip to supply stable 3.3V for the system.

#### System operation

**Fig 4:**
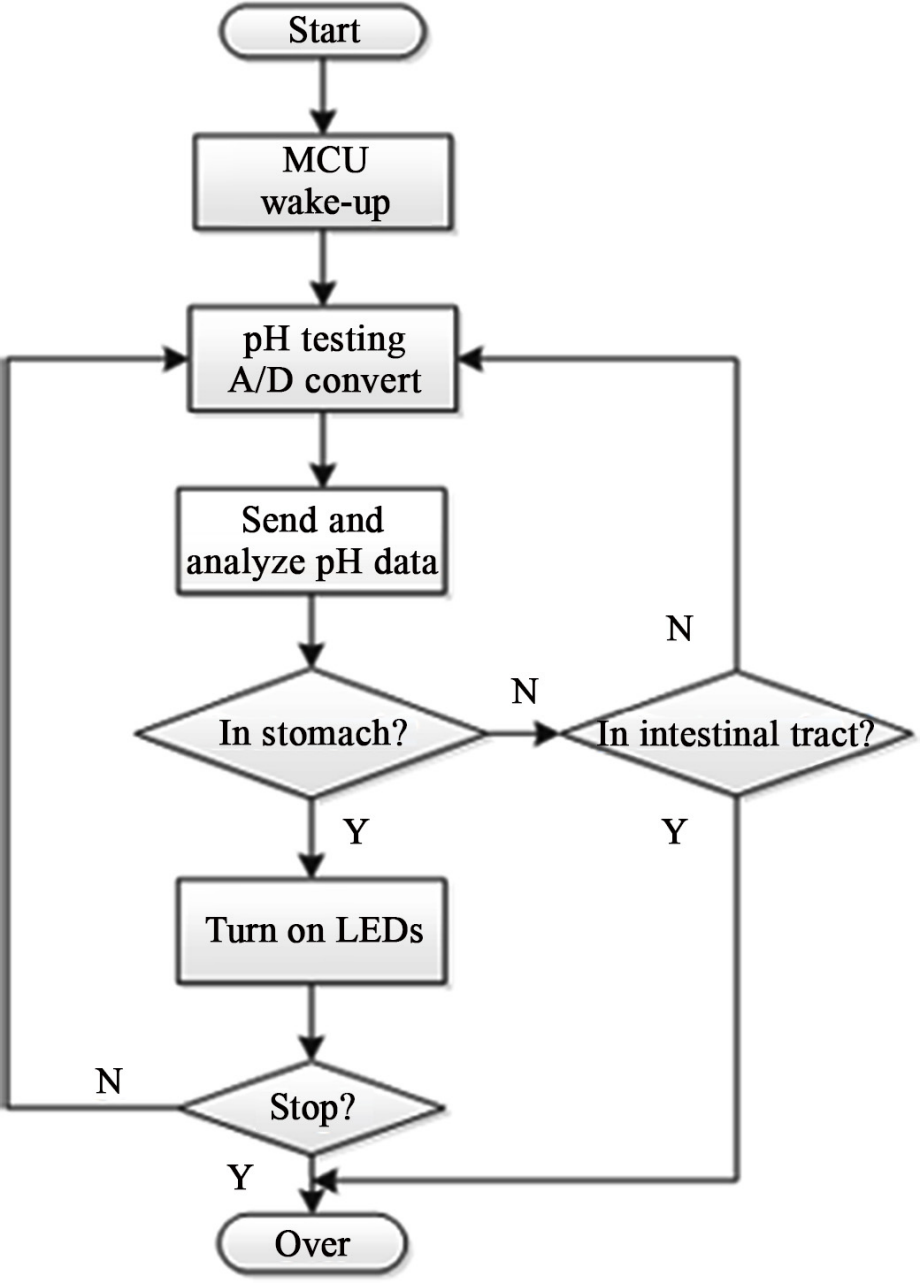
Flow diagram of the system operation.

The capsule is turned on when taken out of the magnetic cover and the MCU enters wake-up mode. The flow diagram of the whole operation is illustrated in Fig 4.

As shown, the system monitors the pH value from the “start” and use analog-to-digital convertor to send out control signal. The pH value is updated every 10 seconds. The MCU processes these pH values to locate the capsule in order to activate other units accordingly. For example, if the capsule moves into the stomach indicated by a pH value of 1~4, the MCU will turn on the LEDs via PWM duty cycle. During this blue-light therapeutic phase (while the capsule is in the stomach), the pH value along with the information on the LEDs status are to be sent to an outside receiver every 10 seconds. Finally, when the pH value indicates that the capsule leaves the stomach and moves into intestinal tract, the LEDs will be turn off, thus the therapy is over and the system enters into the inactive mode.

## Results and Discussions

**Fig 5:**
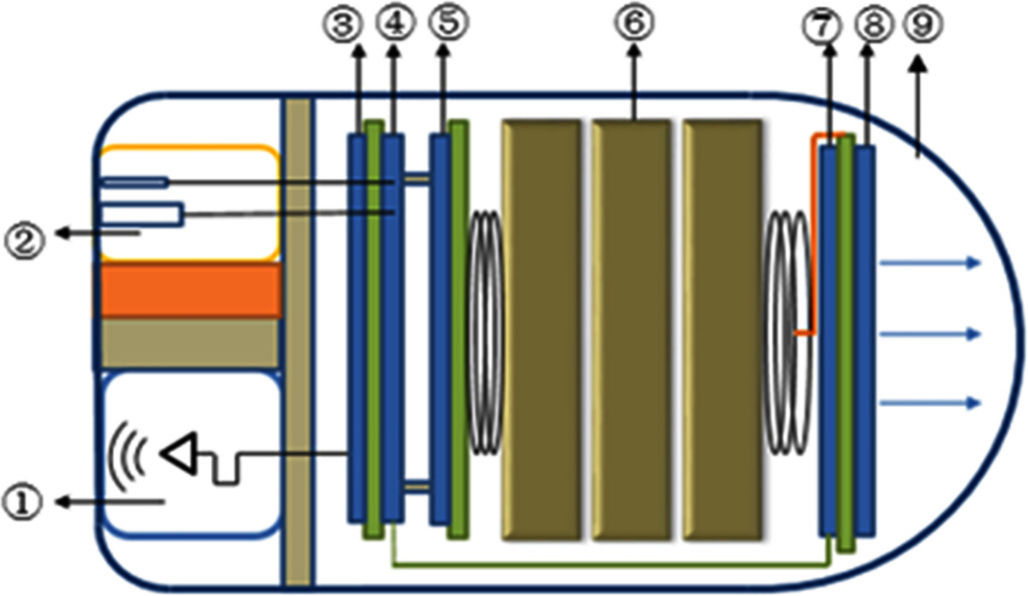
System packaging. ①RF Antenna, ②pH Sensor, ③RF Circuit, ④Micro-controller, ⑤Power Management, ⑥Battery, ⑦LED Driver, ⑧Blue LEDs, ⑨Shell.

All of the units are packaged in a small space, as shown in Fig 5. The diameter and length are 11.5mm and 22mm respectively.

### Experiments of pH testing

**Fig 6:**
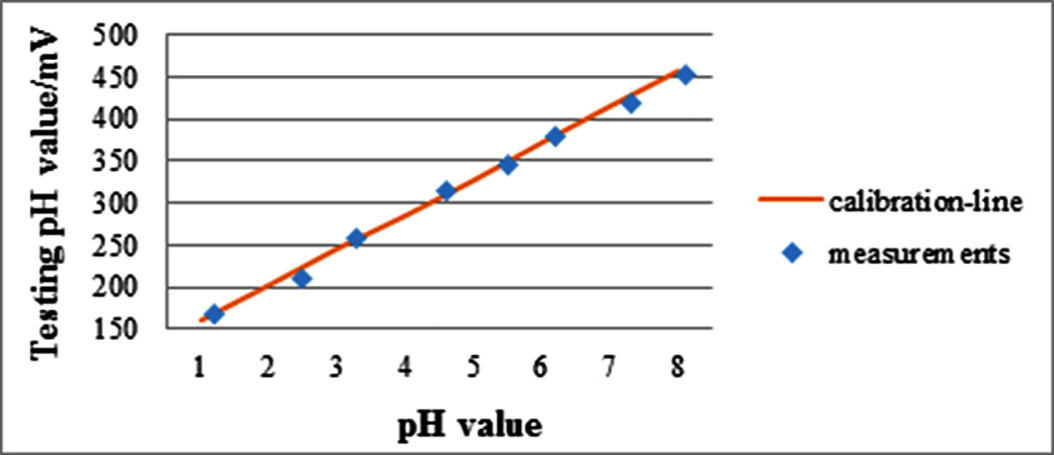
The relationship between the average voltage and pH value measured for the eight testing vessels. This line is used to convert voltage measured by the antimony electrode of the capsule into pH reading.

A series of control experiments are conducted to calibrate the performance of the pH testing unit. Liquid of different acidity with pH value ranging from 1 to 8 is prepared with HCl and NaOH, and then placed in eight vessels labeled as number1 to 8. For each vessel, five repeated experiments were conducted to measure the pH value and the voltage across the two antimony electrodes. Each experiment took about 5 minutes. The pH value was measured by the standard pH meter PB-10 (Sartorius Corporation). Buffer solution with pH value of 7.2 was prepared to calibrate PB-10 and sensor before each measurement [23]. The ambient room temperature is 27°C. The average voltage and pH value over the five replicates were calculated for each vessel, and the results were depicted in Fig 6.

The line shown in Fig 6 is a linear fit relating the voltage to the pH value over a wide range of pH value from 1 to 8. The fit allows us to convert the voltage across electrodes, which is measured by the capsule, into a pH value. The slope of the line, or the measuring sensitivity, is 42mV/pH. The pH value difference between the esophagus and the stomach is about 3, which corresponds to 126mV in voltage. Similarly, the pH value difference between the stomach and the intestinal tract is about 2.6 or 110mV in voltage. The above two voltage thresholds are the significant parameters for the system to detect the capsule location intelligently. It should be noted that the pH testing precision is influenced by temperature. The temperature in the stomach of human is 36.7±0.5°C, and in the intestinal tract is about 36.9°C~37.9°C. Both of them are relatively constant [24–25]. The change in temperature is small, so the temperature influence can be ignored.

### Experiments on blue light therapy unit

The blue LEDs are driven by a current, the higher the current, the higher the blue light intensity and therapeutic effect. The PWM signal controls the current and thus the power of LEDs. Experiment results showed that with a power about 150mW/cm^2^, the blue light could effectively kill *H*. *pylori* [15]. The light irradiation emanates from half spheres at the capsule end. The radius of the sphere is 5.5mm, half the size of PCB board. The capsule comes into contact with the gastric wall and juice. If the contact is with the one of the half spheres, the capsule max power density is about 210mW/cm^2^.

**Fig 7:**
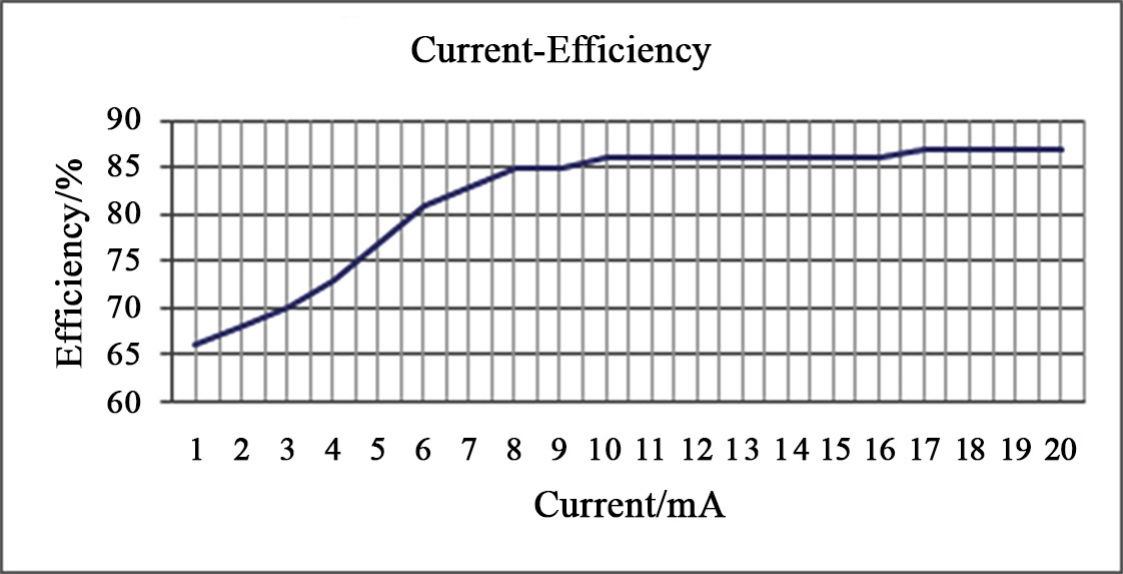
Current efficiency of the therapy unit.

For the first group of experiments, an infinite power source was given and the input voltage was stable at 5V. Experiments on each current were conducted ten times and the interval is one minute. The results are shown in Fig 7.The efficiency of the therapy unit is low until the current reaches up to 17mA, then becomes high and much stabilized.

**Fig 8:**
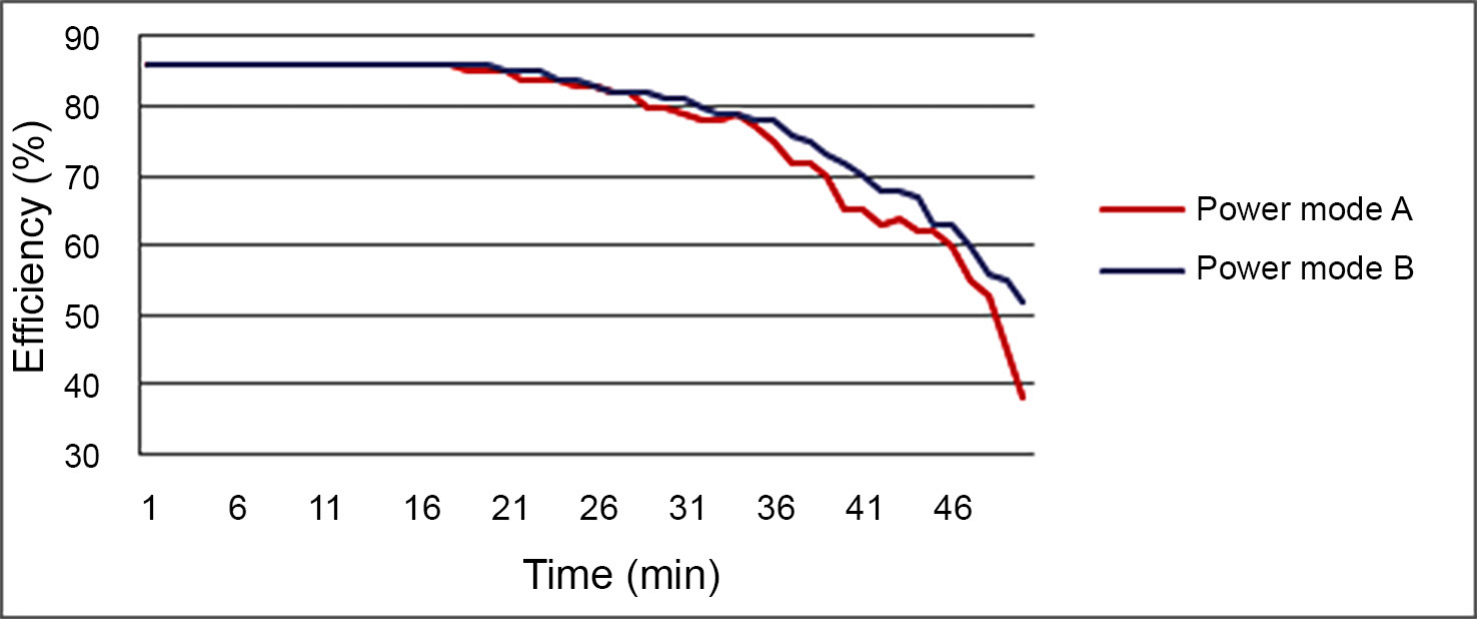
Efficiency of the therapy unit as a function of time for different power modes.

For the second group of experiments, a finite power source is provided by the battery. The input voltage for the unit is the battery output voltage. It reduces with the battery depletion. Experiments were conducted in two power modes (power mode A and B) with the PWM duty cycle set at100% and 85% respectively. The performance of the therapy unit was tested, and the efficiency of this unit is shown in Fig 8 for different power modes. It is should be mentioned that the temperature of the capsule was also tested, and the result shown that it increased in the first 10 minutes and became stable at a safe peak under 43°C.

## Discussions

A blue-light capsule for *H*. *pylori* infection treatment was proposed in this paper. The pH-measuring unit functions as a soft activator for the therapeutic unit as well as a monitoring unit for the digestive tract. Experimental tests on the capsule prototype showed reliable performance for both the pH measuring unit and the LED therapeutic unit. The power of LEDs and the effective area of irradiation are some of the most important factors to impact the therapeutic effectiveness of the capsule. The therapy power density and delivery time are effective. Based on an overall analysis of the performances shown in Figs 7 and 8, the therapy power can be flexible from min mode B to max mode A. The input voltage dropped, and voltage decreased by nearly 15% in 40 minutes, the power of therapy unit also decreased by 15% when working at power mode A. If in power mode B instead, the voltage was more stable and lasted longer than 30 minutes, and the power for the therapy unit is about 176mW/cm^2^. Five minutes delivery with the power density 110mW/cm^2^ was sufficient to reduce the bacterial viability by 99% [15].

It is known that the size and shape of a human stomach change with its state of filling and the body position, which will inevitably impact the effect of irradiation. Under a fasting condition, for which our device is to be deployed, the stomach shrinks down to a small cavity with a diameter comparable to that of intestine. On one hand, this may lead to inaccessible areas such as folds and grooves of its mucosal layer for irradiation treatment. On the other hand, the small cavity makes more feasible to kill *H*. *pylori* locally for the mucosal wall that the device comes in close proximity to. The current device does more for reduction than for eradication of *H*. *pylori*. It is the aim of this paper to initiate the exploration of new methods for phototherapy that may someday achieve the ultimate goal of *H*. *pylori* eradication without antibiotics treatment.

Future experiments will test the *in vivo* efficacy of the capsule in killing *H*. *pylori* in human body. Other design improvements will center on reducing the capsule size, increasing the battery capacity including possibly wireless charging, and optimizing the materials for building the capsule.
